# The cruciferous *Diplotaxis simplex*: Phytochemistry analysis and its protective effect on liver and kidney toxicities, and lipid profile disorders in alloxan-induced diabetic rats

**DOI:** 10.1186/s12944-017-0492-8

**Published:** 2017-05-30

**Authors:** Hamida Jdir, Rihab Ben Abdallah Kolsi, Sami Zouari, Khaled Hamden, Nacim Zouari, Nahed Fakhfakh

**Affiliations:** 1 Laboratory of Enzyme Engineering of Lipases and Biochemistry, Engineering National School of Sfax (ENIS), University of Sfax, Sfax, Tunisia; 2 Laboratory of Animal Ecophysiology, Faculty of Sciences of Sfax, University of Sfax, Sfax, Tunisia; 3 Laboratory of Medicinal and Environmental Chemistry, High Institute of Biotechnology of Sfax, University of Sfax, Sfax, Tunisia; 4 High Institute of Applied Biology of Medenine, University of Gabes, Medenine, Tunisia; 5 Laboratory of Enzyme Engineering and Microbiology, ENIS, University of Sfax, Sfax, Tunisia

**Keywords:** *Diplotaxis simplex*, Glycosylated flavonoids, Diabetic rats, Oxidative stress, Liver, Kidney, Pancreas, Lipid profile

## Abstract

**Background:**

Type 2 diabetes mellitus is a prevalent systemic disease affecting an important proportion of the population worldwide. It has been suggested that excessive reactive oxygen species generation and therefore development of an oxidative stress status is a key factor leading to diabetic complications. Accordingly, it seems that medicinal plants can offer a wide range of new antidiabetic drugs. *Diplotaxis simplex* (Viv.) Spreng. (Brassicaceae) is an edible plant largely distributed in the Mediterranean region. *D. simplex* flowers display important in vitro antioxidant potential and inhibitory activity of the α-glucosidase, a key enzyme linked to type 2 diabetes mellitus. In this paper, the antihyperglycemic potential of *D. simplex* flowers on diabetic rats were investigated.

**Methods:**

Bioactive substances were determined by liquid chromatography-high resolution electrospray ionization mass spectrometry (LC-HRESIMS) analysis. Animals were divided into four groups of six rats each: a normal control group, a diabetic control group, a diabetic group receiving flowers extract (200 mg/kg body mass) and a diabetic group receiving acarbose (10 mg/kg body mass) as standard drug.

**Results:**

Many glycosides of rhamnetin, isorhamnetin, quercetin and kaempferol compounds were identified in the ethanolic flowers extract. Alloxan induced hyperglycemia, manifested by a significant (*p* < 0.05) increase in the blood glucose level as well as in serum α-amylase activity. Furthermore, diabetic rats exhibited oxidative stress, as evidenced by a decrease in antioxidant enzymes activities and an increase in lipid peroxidation level of the pancreas, liver and kidneys. Interestingly, the oral administration of *D. simplex* flowers extract during 30 days restored the glycemia, α-amylase activity, serum lipid profile and antioxidant enzymes. Moreover, the flowers extract exhibited a renal protective role by decreasing the urea and creatinine levels in diabetic rats serum.

**Conclusions:**

*D. simplex* flowers contained bioactive compounds that possess important antioxidant and hypoglycemic properties and protected pancreas, liver and kidneys against hyperglycemia damage.

## Background

Type 2 diabetes mellitus (T2DM), characterized by persistent hyperglycemia, is a chronic metabolic disorder resulting from defects in insulin secretion and/or its action. It represents a major public health problem in all countries around the world. The diabetes prevalence has been steadily increasing for the last three decades and is growing more rapidly in low- and middle-income countries due to the increased factors that lead to T2DM such as overweight and obesity. The World Health Organization [1] estimates that 422 million adults, representing 8.5% among the population, were living with diabetes in 2014. Hyperglycemia is one of the crucial factors responsible for reactive oxygen species (ROS) generation and therefore the development of an oxidative stress status. In fact, oxidative stress is considered as the pathogenic mechanism linking insulin resistance, β- and endothelial cells dysfunction, and eventually leading to T2DM and its complications, such as cardiovascular disease, nephropathy, neuropathy and retinopathy [2, 3]. In 2012 alone diabetes caused 1.5 million deaths [1].

Liver and kidneys are considered as the major organs involved in the xenobiotics excretion. The liver plays a crucial role in the elimination and biotransformation of toxic chemicals. During detoxification, ROS are generated within hepatocytes causing hepatotoxicity or liver damage [4]. Moreover, the kidneys are highly vulnerable to oxidative stress damage, likely due to the abundance of polyunsaturated fatty acids in the renal lipids composition. In fact, ROS are involved in the pathogenic mechanisms such as glomerulosclerosis and tubulointerstitial fibrosis [5]. Thus, liver and kidneys’ protection remains the major clinical challenge including the development of new substances of herbal origin that can be considered as a useful, safe and effective co-supplement to prevent these vital organs from toxicity risks.

To be protected against the ROS toxic effects, the organisms provide enzymatic and non-enzymatic antioxidants defense systems. Superoxide dismutase (SOD), catalase (CAT) and glutathione peroxidase (GPX) are the major antioxidant enzymes. SOD is responsible for fast conversion of superoxide radicals to H_2_O_2_ and is considered as the first enzyme involved in the detoxifying process, while CAT eliminates H_2_O_2_ by its conversion into H_2_O and O_2_. GPX is also considered to be a powerful ROS scavenger by its broader substrate specifications and strong affinity for H_2_O_2_ [6]. Our antioxidant system is incomplete without the exogenous reducing compounds such as vitamin C, vitamin E, carotenoids and particularly phenolic compounds that quench the excess of ROS. Thus, dietary antioxidants play a key role in reinforcing the cellular antioxidant system. Accordingly, there is a growing interest in using new natural antioxidants to prevent metabolic disorders where oxidative stress is the key factor in health physiopathology [7].

Pancreatic α-amylase and α-glucosidase are responsible for the polysaccharides conversion into glucose that enters in the blood stream. The inhibition of these enzymes is considered as a therapeutic approach to diabetes. In fact, the reduction in carbohydrates digestion reduces the glucose absorption rate and thus decreases the blood glucose level [8]. Some inhibitors such as miglitol, voglibose and acarbose are currently used in clinical practice. Nevertheless, these drugs have some side effects such as diarrhea, flatulence, bloating and abdominal discomfort [9]. Therefore, the search of more safer, specific and effective hypoglycemic agents is in continuous interest. In particular, medicinal plants can offer a wide range of new antidiabetic drugs [10]. Regardless their origin, natural substances show various physiological and therapeutic benefits and they are considered as a natural gift for human health promotion. In this context, many investigations reported that plant extracts may provide a preventive effect against diabetes and its complications in animal models [3].

*Diplotaxis simplex* (Viv.) Spreng. (Brassicaceae) is an edible plant largely distributed in the Mediterranean region. This herb is appreciated for its strong pungent flavour and it is consumed raw or cooked, in salads and soups. In our previous work, *D. simplex* flowers were shown to present an interesting in vitro inhibition of α-glucosidase activity and effectively reduce the rise in blood glucose level of maltose-loaded mice as compared to the standard acarbose [11]. Moreover, *D. simplex* flowers exhibited important antioxidant potential as determined by various complementary methods [12]. However, there are no studies on the bioactive substances identification in *D. simplex* flowers and their hypoglycemic effect in diabetic rats. Therefore, the aim of the present work is to assess the hypoglycemic effect of *D. simplex* flowers in alloxan-induced diabetic rats. The effect of *D. simplex* flowers ethanolic extract (DSEE) on antioxidant enzyme activities, lipid peroxidation, liver and kidney toxicities, and serum lipid profile was investigated. Furthermore, the DSEE was analyzed by Liquid Chromatography-High Resolution Electrospray Ionization Mass Spectrometry (LC-HRESIMS) technique in order to identify the bioactive compounds frequently associated with the antioxidant activity.

## Methods

### Plant material

*Diplotaxis simplex* (Viv.) Spreng. (Brassicaceae) was collected from south-eastern Tunisia (Medenine). A voucher specimen is deposited at the High Institute of Applied Biology of Medenine (Medenine, Tunisia) under the number Ds02. After harvest, the flowers were separated, shade-dried for 20 days, ground into fine powder and then stored in the dark at 4 °C until use.

### Preparation of *D. simplex* extract

The dried powder of the *D. simplex* flowers (25 g) was subjected to Soxhlet-extraction with 300 ml ethanol during 6 h. After that, the solvent was evaporated using a rotary evaporator and then totally removed by flushing with nitrogen. Finally, the *D. simplex* flowers ethanolic extract (DSEE) was kept in the dark at 4 °C until further analysis.

### LC-HRESIMS analysis

One hundred mg of the DSEE was dissolved in 100 ml of 10% methanol, filtered through 0.45 μm filter, and then 1 ml was transferred into LC-MS vials. Reversed-phase column (Pursuit XRs ULTRA 2.8, C18, 100 mm × 2 mm i.d., Agilent Technologies, UK) and a diode array detector were used to carry out HPLC analysis. Twenty μl of the sample were injected into the column set at 30 °C. The Mobile phases consisted of 0.1% formic acid in water (A) and 0.1% formic acid in methanol (B). The gradient program used for separation consisted of 100% solvent A, with a linear gradient of 100% solvent B over 20 min, hold at 100% solvent B for 5 min and 100% solvent A for 25 min. The elution was performed with a rate of 1 ml/min and the drying gas flow rate was 1 ml/min at 320 °C. The mass spectrometer (MS) was operated in the positive ion mode in a mass range of 100–2000 m/z. High resolution mass spectral data were obtained on a Thermo Instruments ESI-MS system (LTQ XL/LTQ Orbitrap Discovery, UK) connected to a Thermo Instruments HPLC system (Accela PDA detector, Accela PDA autosampler and Accela Pump).

### Animals and treatments

Adult Wistar male rats weighing between 160-200 g were obtained from the Central Pharmacy of Tunisia (SIPHAT, Tunis, Tunisia). The animals were maintained under standard environmental conditions of temperature (24 ± 4 °C), relative humidity (45–55%), a 12 h dark/light cycle, with *ad libitum* access to food, pellet diet (SICO, Sfax, Tunisia), and water. The handling of the animals was approved by the Medical Ethics Committee for the Care and Use of Laboratory Animals of the Pasteur Institute of Tunis (approval number: FST/LNFP/Pro 152012) and carried out according to the European convention for the protection of living animals used in scientific investigations [13].

Before initiation of the experiment, the rats were acclimatized for a period of 7 days. Diabetes was induced by a single intraperitoneal injection of alloxan (Sigma-Aldrich, St. Louis, MO, USA) dissolved in 1 ml of 0.9% NaCl and at a dose of 150 mg/kg body mass (BM) [14]. After 2 weeks, rats with blood glucose above 2 g/l were chosen as diabetic animals. A total of 24 rats were divided into four groups, of six animals each, and were subjected to the following treatments during 30 days:

(i) group 1: normal rats considered as non diabetic animals and referred to as “Control”;
(ii) group 2: diabetic control rats referred to as “Diab”;
(iii) group 3: diabetic rats treated with DSEE (200 mg/kg BM) administrated by gastric gavage and referred to as “Diab + DSEE”;
(iv) group 4: diabetic rats treated with acarbose (10 mg/kg BM) administrated by gastric gavage and referred to as “Diab + Acar”.

Four weeks later, the rats were sacrificed by decapitation and their trunk bloods were collected. The serum was prepared by centrifugation (3000 × *g*, 15 min, 4 °C) and then stored at −80 °C until use. In addition, liver, kidney and pancreas were carefully removed, cleaned from fat and then homogenized in 10 mM phosphate buffer pH 7.4, 150 mM NaCl. After centrifugation (5000 × *g*, 20 min, 4 °C), the supernatants were collected and stored at −80 °C until use for biochemical assays.

### Biochemical assays

The analyses of the serum lipid levels of triglycerides (TG), total-cholesterol (T-Ch), high density lipoprotein-cholesterol (HDL-Ch) and low density lipoprotein cholesterol (LDL-Ch) as well as the serum α-amylase, aspartate aminotransferase (AST), alanine aminotransferase (ALT) and lactate dehydrogenase (LDH) activities, urea, creatinine and blood glucose level were performed using commercial kits (Biolabo, Maizy, France) on an automatic biochemical analyzer (Vitalab Flexor E, Spankeren, Netherlands) in the biochemical laboratory of Hedi Chaker Hospital (Sfax, Tunisia).

Lipid peroxidation in the liver, kidney and pancreas of different groups was measured by the quantification of thiobarbituric acid reactive substances (TBARS) determined by the method of Yoshioka et al. [15]. Catalase (CAT) activity was measured as previously described by Mueller et al. [16] and expressed as μmol H_2_O_2_/min/mg protein. Superoxide dismutase (SOD) activity was measured by the method of Marklund and Marklund [17] and expressed as U/mg protein. Glutathione peroxidase (GPX) activity was measured by the method described by Paglia et al. [18] and expressed as μmol GSH/min/mg protein. Protein concentration was determined as previously described [19] using bovine serum albumin (E^1%^_1cm_ = 6.7) as standard.

### Histological analyses

The pancreas, liver and kidneys samples from rats of different treatments were fixed in Bouin solution for 24 h, embedded in paraffin and then sections of 5 μm thickness were stained with hematoxylin-eosin. The slides were photographed with an Olympus U-TU1X-2 camera linked to an Olympus CX41 microscope (Olympus, Tokyo, Japan).

### Statistical analysis

All analytical determinations were performed at least in duplicate for six animals per group. One-way analysis of variance was conducted using the SPSS software for Windows™ (version 17, SPSS Inc., Chicago, IL, USA). Duncan’s multiple range test (*p* < 0.05) was used to compare the average responses between treatments.

## Results and discussion

### Phytochemistry analysis

The results of the phytochemical profile of *D. simplex* flowers ethanolic extract (DSEE) were presented in Table 1. The liquid chromatography-high resolution electrospray ionization mass spectrometry (LC-HRESIMS) analysis of the DSEE allowed the identification of 15 compounds divided into 11 flavonoids, 2 alkaloids and 2 triterpenoids. The flavonoids were mainly represented by glycosides of rhamnetin, isorhamnetin, quercetin and kaempferol (Table 1). A survey of the literature shows that most of the identified compounds had potent antioxidant potential. In fact, the IC_50_ values relative to the DPPH• radical-scavenging activities of compounds **1**, **3**, **4**, **5**, **7**, **8** and **10** were 18, 5.2, 0.8, 50.8, 0.2, 1.7 and 9.2 μg/ml, respectively [20–24]. Moreover, Legault et al. [25] reported that quercetin-7-*O*-β-D-glucopyranoside (compound **9**) exhibit a strong antioxidant potential in the oxygen radical absorbance capacity assay. These results confirmed our previous findings, showing that *D. simplex* flowers contain relatively high flavonoids level (74.2 ± 3.4 mg QE/g extract) and display an interesting antioxidant potential as shown through various antioxidant mechanisms, including the β-carotene bleaching protection (IC_50_ = 12.5 ± 0.02 μg/ml), Fe^3+^ reducing (EC_50_ = 0.10 ± 0.01 mg/ml), DPPH• radical-scavenging (IC_50_ = 0.20 ± 0.02 mg/ml) and Fe^2+^ chelating (IC_50_ = 0.60 ± 0.02 mg/ml) assays as well as through DNA damage protection [12].

**Table 1:** LC-HRESIMS analysis of the *D. simplex* flowers ethanolic extract

Compound NO.	Suggested compounds^a^	Accurate mass	Molecular formula^b^
Flavonoids			
1	Rhamnetin 3,3’-di-*O*-β-D-glucopyranoside	641.16956	C_28_H_32_O_17_
2	Rhamnetin 3-*O*-β-D-galactopyranoside-3’,4’-di-*O*-β-D-glucopyranoside	803.22205	C_34_H_42_O_22_
3	Isorhamnetin	317.06439	C_16_H_12_O_7_
4	Isorhamnetin 7-*O*-β-D-glucopyranoside	479.11688	C_22_H_22_O_12_
5	Isorhamnetin 3-*O*-α-D-rhamnopyranoside	463.12379	C_22_H_22_O_11_
6	Isorhamnetin 3-*O*-β-D-glucopyranoside-4’-*O*-β-D-xylopyranoside	611.15894	C_27_H_30_O_16_
7	Quercetin	303.04963	C_15_H_10_O_7_
8	Quercetin 3-*O*-β-D-glucopyranoside	465.10225	C_21_H_20_O_12_
9	Quercetin 7-*O*-β-D-glucopyranoside	465.10295	C_21_H_20_O_12_
10	Kaempferol 3-*O*-β-D-glucopyranoside	449.10614	C_21_H_20_O_11_
11	Kaempferol 3-*O*-β-D-glucopyranoside-4’-*O*-β-D-xylopyranoside	581.15030	C_26_H_28_O_15_
Alkaloids			
12	Tenualexin	217.09735	C_12_H_12_N_2_O_2_
13	Arvelexin	187.08669	C_11_H_10_N_2_O
Triterpenoids			
14	Iristectorene B	685.58179	C_44_H_76_O_5_
15	Iristectorene G	741.64502	C_48_H_84_O_5_

^a^The compounds were suggested according to the Dictionary of Natural Products (DNP 23.1, 2015 on DVD) and characteristic fragmentation pattern; ^b^The formulas were deduced from the quasimolecular ion peak [M + H]^+^

Previous studies also showed that many of the identified compounds in the DSEE were reported in other cruciferous species and they exhibited interesting biological properties. Indeed, the flavonoids **3**, **4**, **5**, **6**, **7**, **8**, **9**, **10** and **11** were previously reported on aerial parts of *Diplotaxis harra* [26]. Kassem et al. [26] also showed that the pure flavonoids isorhamnetin 7-*O*-β-D-glucopyranoside, isorhamnetin 3-*O*-β-D-glucopyranoside-4’-*O*-β-D-xylopyranoside and kaempferol 3-*O*-β-D-glucopyranoside-4’-*O*-β-D-xylopyranoside (compounds **4**, **6** and **11**, respectively) present antiviral activity against foot-and-mouth disease viruses. Furthermore, the isolated flavonoids isorhamnetin 7-*O*-β-D-glucopyranoside, quercetin, quercetin 3-*O*-β-D-glucopyranoside and kaempferol 3-*O*-β-D-glucopyranoside (compounds **4**, **7**, **8** and **10**, respectively) from aerial parts of *D. harra* showed cytotoxicity against HCT116 cell line with IC_50_ values of 20.1, 24.3, 22.8 and 41.9 μg/ml, respectively [27]. Haraguchi et al. [28] reported that quercetin (compound **7**) and quercetin 3-*O*-β-D-glucopyranoside (compound **8**) exhibit an inhibitory activity of aldose reductase, the principal enzyme of polyol pathway, which plays a vital role in the development of diabetic complications. Indeed, the IC_50_ values relative to aldose reductase activity inhibition of compounds **7** and **8** were found to be 15.2 and 7.4 μg/ml, respectively [28]. Legault et al. [25] also mentioned that quercetin-7-*O*-β-D-glucopyranoside (compound **9**) possesses important anti-inflammatory activity by inhibiting NO release, inducible nitric oxide synthase and cyclooxygenase-2 expression, and granulocyte macrophage colony-stimulating factor overproduction. On the other hand, the flavonol diglycoside rhamnetin 3,3’-di-*O*-β-D-glucopyranoside (compound **1**), that was previously isolated from *Diplotaxis virgata* and *Diplotaxis erucoides*, exhibited an interesting antibacterial activity [23].

In addition to flavonoids, two alkaloids tenualexin (compound **12**) and arvelexin (compound **13**) were identified (Table 1). The tenualexin (2-(1,4-dimethoxy-1*H*-indol-3-yl) acetonitrile) is a cruciferous phytoalexin that was previously isolated from *Diplotaxis tenuisiliqua*. Pedras and Yaya [29] reported that tenualexin appears to be one of the broad-range antifungals occurring in crucifers, which suggesting that *D. tenuisiliqua* could have disease resistance traits of interest in commercial breeding programs. The arvelexin (4-methoxyindole-3-acetonitrile) is a biologically active compound found in *Brassica rapa* (Brassicaceae) that is able to inhibit colonic inflammation by suppressing NF-κB activation in dextran sulfate sodium-induced mice and TNF-α-induced colonic epithelial cells [30]. The anti-inflammatory activity of compounds **9** and **13** was consistent with our previous findings suggesting that *D. simplex* flowers presented anti-inflammatory potential by reducing the paw oedema in mice, 4 h post carrageenan challenge [12].

Table 1 also shows that some compounds were identified for the first time in the cruciferous species such as the triglycoside rhamnetin 3-*O*-β-D-galactopyranoside-3’,4’-di-*O*-β-D-glucopyranoside (compound **2**) that was previously isolated from the aerial parts of *Anthyllis onobrychioides* [31]. Moreover, the monocyclic triterpene esters such as iristectorene B (compound **14**) and iristectorene G (compound **15**) were isolated from the seeds of *Iris tectorum* [32].

### DSEE effect on the blood glucose level and α-amylase activity

**Fig. 1:**
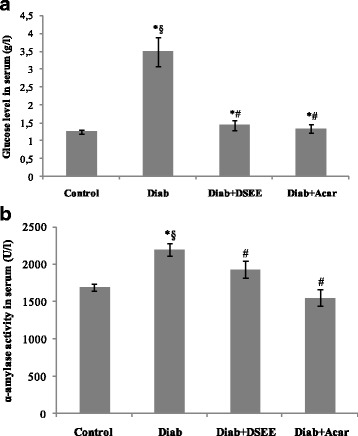
Effect of *Diplotaxis simplex* ethanolic extract (DSEE) on the blood glucose level (**a**) and serum α-amylase activity (**b**). Data represented mean ± SD (*n* = 6 for each group). Values differed significantly at *p* < 0.05. ^*^indicated significant differences as compared to normal rats (Control). ^#^indicated significant differences as compared to diabetic rats (Diab). ^§^indicated significant differences as compared to diabetic rats treated by acarbose (Diab + Acar)

Figure 1 illustrates the blood glucose and serum α-amylase activity levels in animals of different groups. Obtained results showed that oral administration of acarbose, one of the leading inhibitors of carbohydrate metabolic enzymes in the gastrointestinal tract, to alloxan-induced diabetic rats decreased the glycemia by 62% and restored the normal level. Interestingly, this reduction rate was similar to that recorded after *D. simplex* flowers ethanolic extract (DSEE) treatment, suggesting its potential hypoglycemic effect. As compared to the normal rats, a significant (*p* < 0.05) increase by 30% in the serum α-amylase activity was measured in diabetic rats, which represent a marker of pancreas damage. Figure 1b shows that oral administration of acarbose resulted in reducing the serum α-amylase activity by 29% as compared to the untreated diabetic rats. Similarly, DSEE-treatment also reduced the serum α-amylase activity to near the level of control animals. These results are in agreement with several studies dealing with the hypoglycemic effects of various plants on alloxan-induced diabetic rats, such as *Zygophyllum album* extracts [33]. Therefore, it is possible that the observed reduction in serum α-amylase activity by the DSEE treatment was connected to the normal hyperglycemia restoration.

### Evaluation of the antioxidant enzymes activities and lipid peroxidation in liver, pancreas and kidneys tissues

The effect of the DSEE administration on lipid peroxidation and antioxidant enzymes in liver, pancreas and kidneys of diabetic rats was investigated (Table 2). The obtained results showed that antioxidant enzyme activities such as CAT, SOD and GPX decreased in the different organs of diabetic rats when compared to the normal rats, which proved the development of severe oxidative stress status. The inactivation of powerful antioxidant enzymes such as CAT and SOD by diabetic complications was previously described by Yan and Harding [34]. Ceriello and Motz [2] reported that oxidative stress alters the intracellular signaling pathway inducing insulin resistance with reduced intracellular antioxidant defense. More recently, Kumawat et al. [35] showed that oxidative stress extent and the deficiency of defensive antioxidant mechanisms in diabetic patients depend on the occurrence of complications in the diabetes metabolic control. Interestingly, the DSEE administration to the diabetic rats for 1 month significantly (*p* < 0.05) increased the antioxidant enzymes activities, which indicated an antioxidant status improvement. For example, compared to the untreated diabetic animals, the SOD activity raised by 2.24, 2.19 and 3.18 folds in the liver, pancreas and kidneys, respectively. A similar trend was also observed for CAT and GPX activities (Table 2). Table 2 also presents the TBARS content, which gave an idea about the polyunsaturated fatty acids peroxidation level, in all the rat groups. As it can be clearly observed and contrary to diabetic animals, the DSEE treatment significantly (*p* < 0.05) decreased the TBARS content in the liver (80.8%), pancreas (31.6%) and kidney (53.5%) and the values were comparable to the normal rats in control group. It is worthy to note that DSEE effect on antioxidant enzymes and TBARS content was almost equal to that indicated by the standard acarbose. These results suggested the positive effect of DSEE on improving the rat’s antioxidant status by activating its enzymatic antioxidants, and in turn, reducing the lipid peroxidation reactions in vital tissues. The obtained results were in accordance with those got from many plant extracts that exhibited hypoglycemic, hypolipidemic, and antioxidant effects in animals as well as in humans. For instance, Sefi and al. [36] proved that *Centaurium erythrea* extract alleviates streptozotocin-induced oxidative stress and β-cell damage in rat pancreas. These authors reported a significant increase in the activities of both enzymatic and non-enzymatic antioxidants and thereby a significant reduction in the TBARS and blood glucose levels when compared to untreated diabetic rats.

**Table 2:** Antioxidant enzyme activities (CAT, SOD and GPX) and lipid peroxidation (TBARS) in liver, pancreas and kidneys tissues

	Control	Diab	Diab + DSEE	Diab + Acar
Liver				
CAT (μmol H_2_O_2_/min/mg)	2.89 ± 0.46	1.34 ± 0.08^ac^	2.83 ± 0.07^bc^	4.08 ± 0.86^ab^
SOD (U/mg)	9.83 ± 0.51	4.20 ± 0.65^ac^	9.44 ± 0.83^bc^	7.18 ± 0.68^ab^
GPX (μmol GSH/min/mg) (×10^−3^)	0.79 ± 0.09	0.42 ± 0.10^ac^	0.75 ± 0.13^b^	0.90 ± 0.07^b^
TBARS (nmol/mg)	0.10 ± 0.01	0.52 ± 0.10^ac^	0.10 ± 0.01^b^	0.10 ± 0.01^b^
Pancreas				
CAT (μmol H_2_O_2_/min/mg)	5.19 ± 0.07	3.10 ± 0.75^ac^	4.71 ± 0.28^ab^	4.35 ± 0.39^ab^
SOD (U/mg)	7.60 ± 0.71	2.95 ± 0.65^ac^	6.47 ± 0.21^ab^	6.54 ± 0.41^ab^
GPX (μmol GSH/min/mg) (×10^−2^)	1.82 ± 0.08	0.92 ± 0.03^ac^	1.89 ± 0.10^bc^	1.38 ± 0.16^ab^
TBARS (nmol/mg)	0.22 ± 0.01	0.38 ± 0.02^ac^	0.26 ± 0.01^*ab^	0.27 ± 0.01^ab^
Kidneys				
CAT (μmol H_2_O_2_/min/mg)	4.72 ± 0.44	2.28 ± 0.23^ac^	3.30 ± 0.12^ab^	3.22 ± 0.25^ab^
SOD (U/mg)	13.07 ± 0.23	3.81 ± 0.85^ac^	12.13 ± 0.88^bc^	10.40 ± 0.71^ab^
GPX (μmol GSH/min/mg) (×10^−3^)	2.56 ± 0.19	0.76 ± 0.11^ac^	1.29 ± 0.32^ab^	1.28 ± 0.22^ab^
TBARS (nmol/mg)	0.15 ± 0.03	0.43 ± 0.03^ac^	0.20 ± 0.05^b^	0.28 ± 0.10^b^

Data represented mean ± SD (*n* = 6 for each group). Diab + DSEE indicated diabetic rats treated with the *Diplotaxis simplex* ethanolic extract (DSEE). Values differ significantly at *p* < 0.05. ^a^indicated significant differences as compared to normal rats (Control). ^b^indicated significant differences as compared to diabetic rats (Diab). ^c^indicated significant differences as compared to diabetic rats treated by acarbose (Diab + Acar)

On the one hand, the obtained data could be explained by the important antioxidant properties of *D. simplex* flowers since several studies suggest that acute effects of hyperglycemia are counterbalanced by antioxidants [2]. In fact, the observed in vivo antioxidant effect of DSEE was in agreement with (i) the interesting in vitro antioxidant potential of *D. simplex* flowers that was previously reported [12] and (ii) the various identified flavonoids that were known by their potent scavenging activity (Table 1). Thus, the DSEE regular consumption enhanced the fight against oxidative stress that might indirectly correct the diabetic state and restore the homeostasis.

On the other hand, the observed effects of the DSEE treatment could be also explained by its inhibitory potential of the key-enzymes linked to T2DM as was noted by the acarbose treatment. In fact, Jdir et al. [12] demonstrated that *D. simplex* flowers extract shows an important in vitro inhibitory activity of the α-glucosidase (IC_50_ = 0.046 mg/ml). The postprandial hyperglycemia itself produces an oxidative stress so that specific inhibition of intestinal glucose absorption that lowers postprandial hyperglycemia may be expected to reduce oxidative stress [2].

### DSEE effect on hepatic dysfunction parameters and lipid profile

The hepatic parameters levels and lipid profiles of control and treated rats were evaluated (Table 3). Data showed a significant (*p* < 0.05) increase in the activities of AST (62%), ALT (28%) and LDH (57%) in diabetic rats as compared to the control rats, suggesting hepatocellular damage as a result of alloxan toxicity. In addition, Table 4 shows an increase in serum levels of TG (10%), T-Ch (23%) and LDL-Ch (20%) as well as a decrease in HDL-Ch (7%) of diabetic rats as compared to the control group. These observed disorders in diabetic rats may be explained by the oxidative stress damage caused by the diabetogenic agent alloxan. It was also suggested that hepatic damage may cause this abnormal rise in lipid profile parameters and liver enzymes levels [37]. Interestingly, the DSEE administration showed an important protective action in diabetic rats by reducing the hepatic toxicity. Indeed, the DSEE treatment significantly (*p* < 0.05) reduced the AST, ALT and LDH enzymes activities and the obtained values were similar to those of the control rats (Table 3). Furthermore, as shown in Table 4, the serum lipid profile progressed to the normal values of control rats which is in agreement with the research conducted elsewhere [37]. It is also important to note that DSEE effect on the hepatic dysfunction parameters and lipid profile was similar to that obtained by standard acarbose (Tables 3, 4).

**Table 3:** Liver-kidneys toxicities indices

	Control	Diab	Diab + DSEE	Diab + Acar
Liver toxicity indices				
AST (U/l)	170.33 ± 4.60	276 ± 21.27^ac^	199.71 ± 6.84^b^	177.87 ± 3.85^b^
ALT (U/l)	53.16 ± 3.70	68 ± 2.24^ac^	53.83 ± 0.86^b^	51.37 ± 1.81^b^
LDH (U/l)	784.66 ± 75.8	1231.8 ± 115.63^ac^	799.16 ± 52.06^b^	701.66 ± 19.63^b^
Kidneys toxicity indices				
Urea (mmol/l)	5.80 ± 0.22	8.78 ± 0.20^ac^	7.03 ± 0.15^b^	6.91 ± 0.62^b^
Creatinine (μmol/l)	27.57 ± 0.35	31.02 ± 0.60^ac^	26.89 ± 0.38^b^	28.05 ± 0.56^b^

Data represented mean ± SD (*n* = 6 for each group). Diab + DSEE indicated diabetic rats treated with the *Diplotaxis simplex* ethanolic extract (DSEE). Values differ significantly at *p* < 0.05. ^a^indicated significant differences as compared to normal rats (Control). ^b^indicated significant differences as compared to diabetic rats (Diab). ^c^indicated significant differences as compared to diabetic rats treated by acarbose (Diab + Acar)

**Table 4:** Serum lipids levels of triglycerides (TG), total − cholesterol (T-Ch), high density lipoprotein-cholesterol (HDL-Ch) and low density lipoprotein cholesterol (LDL-Ch)

Serum lipid profile	Control	Diab	Diab + DSEE	Diab + Acar
TG (mmol/l)	1.30 ± 0.01	1.43 ± 0.02^ac^	1.32 ± 0.09^b^	1.25 ± 0.03^b^
T-Ch (mmol/l)	1.39 ± 0.04	1.80 ± 0.01^ac^	1.49 ± 0.07^bc^	1.30 ± 0.07^b^
LDL-Ch (mmol/l)	0.60 ± 0.07	0.72 ± 0.02^ac^	0.60 ± 0.05^b^	0.63 ± 0.01^b^
HDL-Ch (mmol/l)	0.41 ± 0.01	0.38 ± 0.01^ac^	0.42 ± 0.02^b^	0.41 ± 0.01^b^

Data represented mean ± SD (*n* = 6 for each group). Diab + DSEE indicated diabetic rats treated with the *Diplotaxis simplex* ethanolic extract (DSEE). Values differ significantly at *p* < 0.05. ^a^indicated significant differences as compared to normal rats (Control). ^b^indicated significant differences as compared to diabetic rats (Diab). ^c^indicated significant differences as compared to diabetic rats treated by acarbose (Diab + Acar)

### DSEE effect on the renal dysfunction indices

Table 3 presents the kidneys toxicity indices of control and treated rats. The obtained results showed that hyperglycemia increased the urea and creatinine levels by 51 and 12%, respectively in diabetic rat serum as compared to control group. Interestingly, the DSEE or acarbose administration to diabetic rats improved the indices related to kidney dysfunction induced by diabetes. In fact, the DSEE administration to diabetic rats significantly (*p* < 0.05) decreased the urea and creatinine levels by 20% and 13%, respectively, which is consistent with previous studies [33]. As compared to normal individuals, high urea and creatinine levels represent important kidney dysfunction markers [38]. Thus, it can be concluded that diabetic rats suffer from renal disorders due to the protein glycation that lead to the muscle loss as well as to the increase of purine release, which is the main uric acid source [39]. The alleviation of kidneys dysfunction parameters could be explained by the attenuation of oxidative stress situation *via* serum glucose level regulation.

### Histological examination

**Fig. 2:**
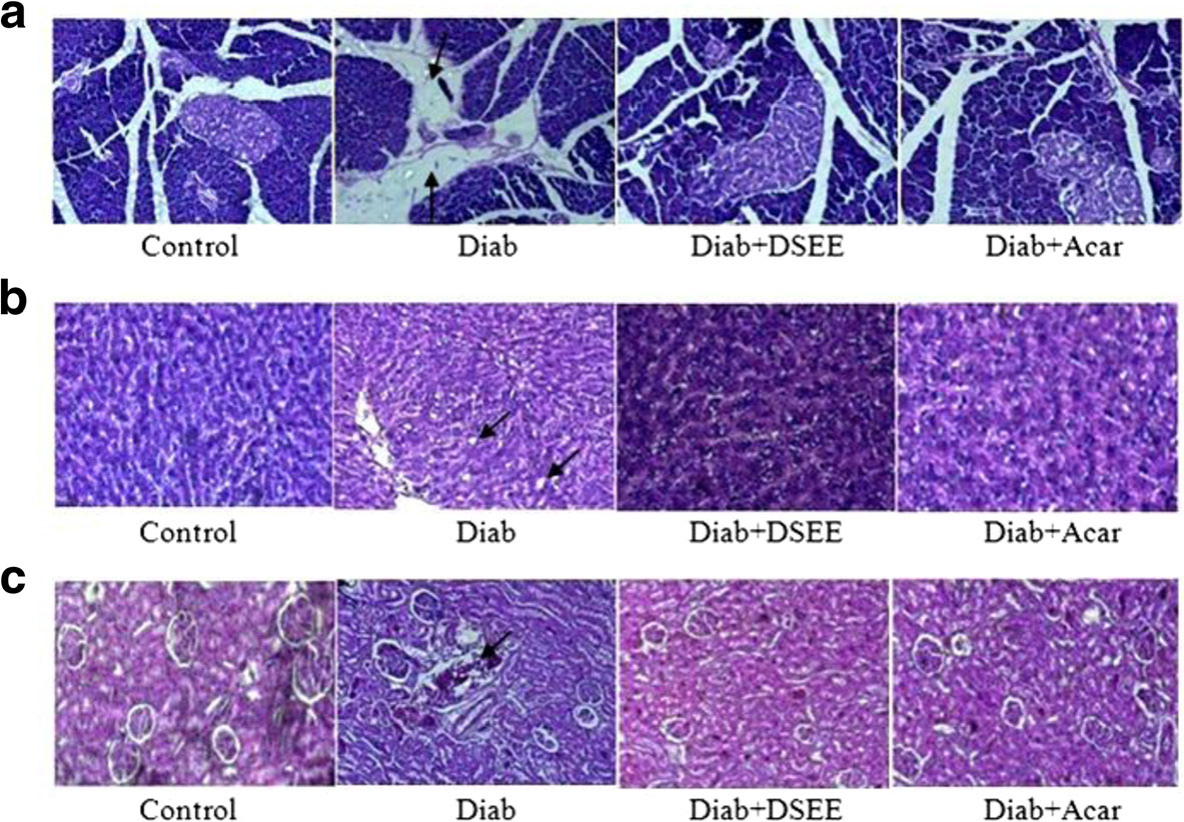
Histological observation of the pancreas (**a**), liver (**b**) and kidneys (**c**) of normal rats (Control), diabetic rats (Diab), diabetic rats treated with *Diplotaxis simplex* ethanolic extract (Diab + DSEE) and diabetic rats treated with acarbose (Diab + Acar). The arrows in the histological sections of the pancreas, liver and kidneys of diabetic rats showed Langerhans islets destruction, lipid accumulation in liver cells and glomerulus condensation, respectively

The findings relative to the DSEE protective effect on alloxan-induced diabetic rats obtained through biochemical assays were further confirmed by histological analysis. Therefore, the effects of DSEE or acarbose treatments on the pancreas (Fig. 2a), liver (Fig. 2b) and kidneys (Fig. 2c) tissues were histologically examined. While normal Langerhans islets with healthy cells were observed in the control rats, a clear destruction in the Langerhans islets of diabetic rats’ pancreas was noted as indicated by the arrows (Fig. 2a). In addition, the histological observations of diabetic rat’s liver revealed an intracellular accumulation of lipids in the hepatocyte cytoplasm as mentioned by the arrows (Fig. 2b). Moreover, the kidneys of the diabetic rats showed an increase in capsular space and glomerular condensation as also displayed by the arrow (Fig. 2c). Interestingly, in DSEE-treated diabetic rats the histological examination showed a protective action on Langerhans islets as compared to untreated diabetic rats. Besides, the DSEE administration to diabetic rats also reduced the appearance of fat cells in the liver and inhibited the glomerular condensation in the kidneys as compared to the untreated diabetic rats. The results also showed that DSEE effect in reducing histopathological injuries was similar to that of acarbose. The histological examination confirmed the importance of the DSEE administration to diabetic rats in alleviating the pancreas, liver and kidneys disorders as demonstrated by the biochemical parameters (Tables 2 and 3). In this context, comparable protective effect on the pancreas, liver and kidneys was obtained using several plant extracts in alloxan-induced diabetic rats [33]. The architecture damages observed in the analyzed tissues were a consequence of the hyperglycemia situation. Thus, DSEE treatment by attenuating hyperglycemia and oxidative stress protected tissues from the deleterious effects caused by diabetes.

## Conclusions

It is evident that diabetic state induces oxidative stress, which contributes to the activation of various downstream signaling cascade causing structural and functional damages in different organs. The present study interestingly revealed that administration of *D. simplex* flowers extract during 30 days protected against alloxan-induced hyperglycemia and limited the extent of the pancreas, liver and kidneys histopathological injuries. Taking into consideration the demonstrated protective potential, these results encourage further exploration of *Diplotaxis simplex* flowers in preventing diseases arising from oxidative damage.
